# The Unfolded Protein Response Plays a Predominant Homeostatic Role in Response to Mitochondrial Stress in Pancreatic Stellate Cells

**DOI:** 10.1371/journal.pone.0148999

**Published:** 2016-02-05

**Authors:** Hsin-Yuan Su, Richard T. Waldron, Raymond Gong, V. Krishnan Ramanujan, Stephen J. Pandol, Aurelia Lugea

**Affiliations:** 1 Pancreatic Research Group, Department of Medicine, Cedars-Sinai Medical Center, Los Angeles, California, United States of America; 2 Department of Medicine, David Geffen School of Medicine, UCLA/VA Greater Los Angeles Health Sciences Center, Los Angeles, California, United States of America; 3 Metabolic Photonics Laboratory, Departments of Surgery and Biomedical Sciences, Cedars-Sinai Medical center, Los Angeles, California, United States of America; University of Valencia, SPAIN

## Abstract

Activated pancreatic stellate cells (PaSC) are key participants in the stroma of pancreatic cancer, secreting extracellular matrix proteins and inflammatory mediators. Tumors are poorly vascularized, creating metabolic stress conditions in cancer and stromal cells that necessitate adaptive homeostatic cellular programs. Activation of autophagy and the endoplasmic reticulum unfolded protein response (UPR) have been described in hepatic stellate cells, but the role of these processes in PaSC responses to metabolic stress is unknown. We reported that the PI3K/mTOR pathway, which AMPK can regulate through multiple inputs, modulates PaSC activation and fibrogenic potential. Here, using primary and immortalized mouse PaSC, we assess the relative contributions of AMPK/mTOR signaling, autophagy and the UPR to cell fate responses during metabolic stress induced by mitochondrial dysfunction. The mitochondrial uncoupler rottlerin at low doses (0.5–2.5 μM) was added to cells cultured in 10% FBS complete media. Mitochondria rapidly depolarized, followed by altered mitochondrial dynamics and decreased cellular ATP levels. This mitochondrial dysfunction elicited rapid, sustained AMPK activation, mTOR pathway inhibition, and blockade of autophagic flux. Rottlerin treatment also induced rapid, sustained PERK/CHOP UPR signaling. Subsequently, high doses (>5 μM) induced loss of cell viability and cell death. Interestingly, AMPK knock-down using siRNA did not prevent rottlerin-induced mTOR inhibition, autophagy, or CHOP upregulation, suggesting that AMPK is dispensable for these responses. Moreover, CHOP genetic deletion, but not AMPK knock-down, prevented rottlerin-induced apoptosis and supported cell survival, suggesting that UPR signaling is a major modulator of cell fate in PaSC during metabolic stress. Further, short-term rottlerin treatment reduced both PaSC fibrogenic potential and IL-6 mRNA expression. In contrast, expression levels of the angiogenic factors HGF and VEGFα were unaffected, and the immune modulator IL-4 was markedly upregulated. These data imply that metabolic stress-induced PaSC reprogramming differentially modulates neighboring cells in the tumor microenvironment.

## Introduction

Activated pancreatic stellate cells (PaSC) are the main cell type in the stroma of chronic pancreatitis and pancreatic cancer and participate in the progression of these disorders [1, 2]. After pancreas damage [3] and in the fibrotic stroma, quiescent PaSC become “activated” and differentiate into a myofibroblast phenotype that synthesizes and secretes large amounts of extracellular matrix proteins, as well as various cytokines and growth factors. These factors are critical for buildup of stroma, and exert autocrine and paracrine effects on PaSC and neighboring cells [1, 4]. Since their identification in 1998 [5, 6], research has focused on understanding how growth factors and cytokines, and intracellular downstream signaling govern PaSC activation. However, little is known about the role of homeostatic cellular programs including autophagy and endoplasmic reticulum (ER) signaling in PaSC reprogramming during activation and under metabolically stressful conditions such as that within a poorly vascularized stromal microenvironment.

Stellate cell activation is accompanied by rapid cell growth, proliferation, and expansion of the mitochondria and endoplasmic reticulum (ER) networks to meet the bioenergetic and biosynthetic demands of the newly acquired secretory phenotype [1]. These activities are supported by a balance between PI3K/AKT/mTOR signaling and autophagy to cope with a high demand for energy [2, 7, 8]. Autophagy is a cellular catabolic mechanism responsible for recycling of organelles, proteins and lipids, thereby helping to maintain cellular homeostasis and provide substrates for energy production. In conditions of metabolic stress, autophagy allows cells to restore energy generation and promotes survival [9]. Autophagy is required for many physiological processes, and its impairment is often apparent in pathologic states [10]. In a recent study, autophagy-deficient hepatic stellate cells failed to acquire the activated state and displayed a reduced secretory phenotype [8]. These data suggested that autophagy may modulate PaSC remodeling in the progression from a quiescent to an activated phenotype, and/or favor conversion to a secretory phenotype. In this respect, recent data indicate that mTOR and autophagy are key regulators of cellular reprogramming [11] and the hypersecretory phenotype of senescent cells [11, 12], supporting a role for these cellular programs in PaSC reprogramming.

Besides autophagy, the unfolded protein response (UPR) signaling is another important homeostatic regulatory mechanism. The UPR is activated when unfolded/misfolded proteins accumulate in the ER lumen. An adaptive UPR helps to maintain ER homeostasis by adjusting ER protein folding and lipid synthesis demands to the bioenergetics and capacity of the ER [13]. The UPR also modulates dynamic interactions between ER and mitochondria that support ER function. This interaction comprises several processes including ATP influx into the ER, and regulation of mitochondrial dynamics and autophagy [14, 15]. Upon diverse cellular stresses, the UPR can trigger proapoptotic signaling downstream of the ER-transmembrane sensor PKR-like ER kinase (PERK). Short-term PERK activation inhibits general protein translation by catalyzing phosphorylation of eukaryotic initiation factor 2-α (eIF2α) at Ser51, while persistent PERK activation leads to upregulation of the proapoptotic transcription factor C/EBP homologous protein (CHOP) [16]. CHOP is required for ER stress-induced apoptosis [17], which is promoted by diverse mechanisms including CHOP-induced transcription of death receptor 5 (DR5) [18], dysregulation of autophagic regulators including p62/SQSTM1 [19], and cellular ATP depletion linked to CHOP-induced increases in ER protein translation [20].

Since ER protein folding requires high energy in the form of ATP, UPR activation can be an indicator of low cellular energy status [21]. The integration of the UPR and autophagy with sensors of cellular metabolism may be critical for PaSC and tumor cells to withstand and adapt to environmental stresses such as nutrient deprivation, hypoxia and oxidative stress. AMP-activated kinases (AMPK) are key cellular energy sensors, becoming activated by conditions that deplete cellular ATP and elevate AMP levels, such as glucose deprivation or mitochondrial uncoupling [22]. AMPK actions promote energy production through regulation of cellular metabolism and attenuated energy utilization to ensure cellular survival, growth and proliferation. Negative regulation of mTOR complex 1 (mTORC1) and associated anabolic processes, along with autophagy and apoptosis induction, are hypothesized to mediate downstream effects of AMPK [23].

Given the putative role of PaSC in neoplasia, identification of agents that differentially modulate autophagy and ER stress responses is important in developing a clinical armamentarium to modulate PaSC or their phenotypic expression. Previously, our group showed that rottlerin, a phytochemical from the kamala tree, caused mitochondrial stress and reduced pancreatic tumor volume in an orthotopic model of pancreatic cancer [24, 25]. Other studies illustrated the capacity of rottlerin to depolarize mitochondria [24, 26, 27], and showed this was accompanied by activation of the metabolic sensor AMPK and disordered autophagy [28, 29]. Here, we used cultured PaSC treated with rottlerin as a model to examine how metabolic stress caused by mitochondrial dysfunction and ATP depletion alter autophagic and UPR signaling, cell fate, and fibrogenic and inflammatory responses in PaSC.

## Materials and Methods

### Antibodies and chemicals

The following antibodies were used for Western blotting and immunofluorescence: Fibronectin (#F3648; Sigma-Aldrich), GAPDH (#9484; Abcam), LAMP-2 (#L0668; Sigma-Aldrich), Tom20 (FL145, #sc11415; Santa Cruz Biotechnology) and α-SMA (#A2547; Sigma-Aldrich). Antibodies directed against phospho-p70 S6 Kinase (Thr389; #9234), p70 S6 Kinase (#2708), phospho-S6 ribosomal protein (Ser240/244; #5364), S6 ribosomal protein (#2317), phospho-AMPKα (Thr172; #2535), AMPKα (#2532), phospho-eIF2α (Ser51; #3597), eIF2α (#9722), phospho-4E-BP1 (Thr37/46; #2855), CHOP (#5554; for Western blotting), CHOP (#2895; for immunofluorescence); p44/42 MAPK (Erk1/2; #9102), Caspase-3 (#9665), Bim (#2819), LC3B (#2775), p62/SQSTM1 (#5114), Beclin-1 (#3738), Bcl-2 (#2876), and corresponding HRP-linked secondary antibodies were from Cell Signaling Technology.

Rottlerin (#R5648), Bafilomycin A1 (#B1793), Pepstatin A (#P5318), and E-64d (#E8640) were from Sigma-Aldrich. MitoTracker Red CMXRos (#M-7512), 4',6-diamidino-2-phenylindole (DAPI), SuperSignal™ West Pico (or Femto) Chemiluminescent Substrate reagent, ProLong Gold antifade mounting medium, and fluorescence-conjugated secondary antibodies were from ThermoFisher Scientific.

Tissue digestion for PaSC isolation was performed using Pronase (#165921), Collagenase P (#1213873) and DNase I (#10104159001), all obtained from Roche. Density gradients for PaSC separation were prepared using Nycodenz (#AN1002423; Accurate Chemical & Scientific Corp), Gey’s balanced salt solution (GBSS; #G9779; Sigma-Aldrich) and bovine serum albumin fraction V (BSA; #03116956001; Roche). Cell culture DMEM/F12 medium (#11330–032) and L-Glutamine (#25030–081) were from ThermoFisher Scientific; antibiotics/antimycotics (1% Penicillin-Streptomycin; #25030–081) and fetal bovine serum (FBS; #FB11) were from Omega Scientific. All chemicals and kits were used according to the manufacturer’s recommendations, unless otherwise indicated.

### Cell culture

Primary mouse PaSC (mPaSC) were obtained from pancreas tissues from C57BL/6 wild-type mice (Harlan), GFP-LC3 (green fluorescent protein-microtubule-associated protein 1 light chain 3) transgenic mice [30] (C57BL/6 background; donated by Dr. Gukoskaya and Dr. Mareninova), or *Chop-/-* mice [31] (purchased at The Jackson Laboratory and backcrossed into the Harlan C57BL/6 background). GFP-LC3 mPaSC express GFP-LC3 fusion protein and were used to facilitate visualization of autophagic puncta formation in live cell microscopy experiments (described below). *Chop-/-* mPaSC were used to gain insights into the role of the transcription factor CHOP in rottlerin-treated cells. Animal studies were approved by the Institutional Animal Care and Use Committee in accordance with the NIH Guide for the Care and Use of Laboratory Animals. The protocols were approved by the Institutional Animal Care and Use Committee of the VA Greater Los Angeles Health Sciences Center (Protocol Number: 02009–04) and the Institutional Animal Care and Use Committee of the Cedars-Sinai Medical Center (Protocol Number: IACUC005232).

PaSC were isolated as previously described [6] with minor modifications. Briefly, pancreata from 1–2 mice were excised, minced and digested in GBSS containing 140 μg/ml pronase, 400 μg/ml collagenase P and 8.75 units/ml DNase I. The cell suspension was filtered through a 100 μm nylon cell strainer (Falcon, Corning) and washed in GBSS supplemented with 0.3% BSA. Then, PaSC were separated by Nycodenz density gradient centrifugation. The Nycodenz gradient was prepared by layering the cell suspension beneath 6 ml of 0.3% BSA/GBSS in a polycarbonate centrifuge tube, and then centrifuged for 20 minutes at 1400 g. Quiescent PaSC were collected from a fuzzy band at the interface near the top of the gradient, and activated and expanded in culture up to passage 2. Activated PaSC were characterized by the presence of α-SMA stress fibers, high production of fibronectin and reduced expression of GFAP.

Immortalized mouse PaSC (imPaSC) were obtained from Dr. Raul Urrutia [32]. Both primary mPaSC and imPaSC were grown in DMEM/F12 media supplemented with 15% FBS, 2 mM L-glutamine, and antibiotics/antimycotics, in a humidified 5% CO_2_ atmosphere. For experimental purpose, cells were seeded in 60-mm dishes in complete DMEM/F12 media supplemented with 10% FBS, and treated with the experimental agents while at 60–80% confluency. Most of the data presented here was obtained from primary mPaSC, and additional supporting experiments including siRNA transfection, oxygen consumption and mRNA expression were performed in imPaSC.

### MTT assay

MTT (Thiazolyl Blue Tetrazolium Bromide) assay was used to measure cellular metabolic activity in mPaSC and imPaSC. Cells were seeded in 24-well plates at 1x10^4^ cells per well. After the indicated treatments in triplicates, MTT was added to the culture medium to yield a final concentration of 0.5 mg/ml, and cells were then incubated for 3 h at 37°C in CO_2_ incubator. After removing medium, DMSO was added to dissolve the insoluble formazan. Absorbance was measured at 595 nm using a plate absorbance reader (SpectraMax M3, Molecular Devices).

### ATP luminescence assay

PaSC were seeded in 60-mm dishes and treated in duplicates as indicated for 30 or 60 minutes. After treatment, total cellular ATP levels were measured using a bioluminescence ATP Determination Kit (#A22066, ThermoFisher Scientific) according to the manufacturer’s recommendations.

### Oxygen consumption assay

The rates of mitochondrial oxygen consumption (OCR) in live imPaSC were measured using a Clark-type oxygen electrode (Strathkelvin Instruments) in a closed-cell respirometric design [33]. Briefly, cells grown in complete DMEM/F12 media supplemented with 15% FBS were trypsinized and re-suspended in phosphate-buffered saline (PBS). After the basal respiration rate was recorded, vehicle or rottlerin was added to the cells, data were collected for a further 15 min and the change in OCR was calculated from the raw OCR data as described [33].

### DNA fragmentation assay

PaSC were plated in 60-mm dishes and treated with different concentrations of rottlerin. Apoptosis was estimated by measuring internucleosomal DNA fragmentation in media and cell lysates using Cell Death Detection ELISAPLUS kit (#11774425001, Sigma-Aldrich) according to the manufacturer’s recommendation.

### Immunoblotting

For immunoblotting experiments, cells were seeded in 60-mm culture dishes and grown in complete 15% FBS media. At 60–80% confluency, culture media was replaced with fresh 10% FBS media and 2 h later cells were treated as indicated. Following treatments, cells were harvested and lysed in RIPA buffer (20 mM Tris-HCl (pH 7.4), 1% Triton X-100, 1% sodium deoxycholate and 0.1% SDS) supplemented with protease and phosphatase inhibitors (cOmplete^TM^ ULTRA and PhosSTOP^TM^, Roche). Equal amount of proteins were loaded onto Novex^®^ 4–20% Tris-Glycine Gels (Life Technologies), and proteins were electrophoretically transferred onto nitrocellulose membranes using Trans-Blot^®^ Turbo^TM^ Transfer Pack and Trans-Blot^®^ Turbo^TM^ Transfer System (Bio-Rad). Membranes were incubated with primary and secondary antibodies, and then proteins were detected with chemiluminescence reagents using PXi Touch Imaging System (Syngene).

### Live cell microscopy

Live control- or rottlerin treated GFP-LC3 mPaSC grown on fluorodish culture dishes (World Precision Instrument) were imaged with an inverted Nikon Eclipse TE2000-S fluorescence microscope equipped with a CoolSnap camera (Roper Scientific). For quantification of GFP-LC3 puncta, cells displaying >10 brightly fluorescent GFP-LC3 puncta were counted as positive. For assessment of mitochondria membrane potential, treated GFP-LC3 cells were loaded with MitoTracker Red CMXRos and imaged as indicated above. Images were analyzed using the MetaMorph imaging system (Universal Imaging Corporation).

### Immunofluorescence analysis

PaSC were grown on glass coverslips until reaching 60% confluency and treated as above. After treatment, cells were fixed with 4% paraformaldehyde and permeabilized with 0.1% Triton X-100 in PBS. After blocking with PBS containing 1% BSA and 0.01% Tween 20, immunostaining was performed by overnight incubation with the appropriate primary antibodies. The cells were then stained with Alexa Fluor 488 or Alexa Fluor 594 conjugated secondary antibodies (Molecular Probes) and 4’6’-diamidino-2-phenylindole (DAPI) as nuclear counterstain. Images were captured using an inverted Nikon Eclipse TE2000-S fluorescence microscope and analyzed using the MetaMorph imaging system (Universal Imaging Corporation).

### siRNA transfections

imPaSC were seeded in 60-mm dishes and cultured overnight in DMEM/F12 media supplemented with 10% FBS. Cells were subsequently transfected with appropriate siRNAs mixed with Lipofectamine^®^ 3000 Transfection Reagent (Life Technologies) according to the manufacturer’s recommendations. Cells were treated with the experimental agents at 48 h post-transfection. The following siRNA products obtained from GE Healthcare/Dharmacon were used: non-targeting Pool control siRNA (#D-001810), AMPKα1 siRNA (#L-041035), AMPKα2 siRNA (#L-040809).

### Real-time PCR analysis

For mRNA expression analysis, cells were collected in buffer RLT (Qiagen) and RNA extracted using the RNeasy^®^ Plus Mini Kit (#74034; Qiagen). Reverse transcription was performed with the iScript Reverse Transcription Supermix (#170–8840; Bio-Rad) using 1 μg of total RNA, and the synthesized cDNA samples were used as templates for quantitative real-time PCR (qPCR) analysis. Kits were used according to the manufacturer’s instructions. All reactions were performed using the Bio-Rad CFX Connect^TM^ Real-Time PCR Detection System and the amplifications were done with the iTaq™ Universal SYBR^®^ Green Supermix (Bio-Rad). The following gene-specific oligonucleotide primers were used: *p62* forward, 5’-AGCTGCCCTCAGCCCTCTA-3’, and reverse, 5’-GGCTTCTCTTCCCTCCATGTT-3’; *Dr5* forward, 5’-TGCAGAGAGGGTATTGACTA-3’, and reverse, 5’-ACACACCGTATTTGTGGTTA-3’; *Col1a1* forward, 5’-TAGGCCATTGTGTATGCAGC-3’, and reverse, 5’-ACATGTTCAGCTTTGTGGACC-3’; *Hgf* forward, 5’- CTTTTTGCCTTCGAGCTATC-3’, and reverse, 5’- GGTCATGCATTCAACTTCTG-3’; *Vegfα* forward, 5’- GACTATTCAGCGGACTCAC-3’, and reverse, 5’- GCACGATTTAAGAGGGGAAT-3’; *Il-6* forward, 5’-CGTGGAAATGAGAAAAGAGTTGTG-3’, and reverse, 5’-CCAGTTTGGTAGCATCCATCATTTCT-3’; *Il-4* forward, 5’-AGCTATTGATGGGTCTCAAC-3’, and reverse, 5’-CTGTGACCTCGTTCAAAATG-3’; m18s forward, 5'-AGTCCCTGCCCTTTGTACACA-3', and reverse, 5'-CGATCCGAGGGCCTCACTA-3'. Relative transcript levels were calculated using the comparative 2^-ΔΔCt^ method [34] and normalized to the housekeeping gene, 18S rRNA.

### Statistical analyses

All experiments were performed in triplicate unless otherwise stated. Data are presented as mean ± SEM. Data were subjected to two tailed Student t-test for comparison between 2 groups and two way analysis of variance (ANOVA) followed by post-hoc Tukey tests for comparisons between more than two groups. P values <0.05 were considered significant.

## Results

Studies using hepatic stellate cells indicate that autophagy and ER stress signaling modulate differentiation from the quiescent to the activated phenotype [8, 35]. Like hepatic stellate cells, pancreatic stellate cells (PaSC) undergo major metabolic changes upon activation, and likely rely on autophagy and the ER unfolded protein response (UPR) to meet the bioenergetic and biosynthetic demands of their acquired secretory phenotype and rapid proliferation. How these cellular homeostatic processes regulate the phenotype of activated PaSC has not been investigated. We hypothesize that activated PaSC in the microenvironment of pancreatic solid tumors experience metabolic stress. In this context, homeostatic cellular programs including autophagy and ER stress signaling modulate the stellate cell activated phenotype, pro- or anti-neoplastic responses and, ultimately cell fate.

In this study we sought to gain insight into the contribution of homeostasis dysregulation in cultured activated mouse PaSC to cell fate decisions and fibrotic and inflammatory responses. To modulate cellular energy status, we treated mouse PaSC grown in 10% FBS media with the mitochondrial uncoupler, rottlerin. We and others previously showed that rottlerin, at concentrations ranging from 2.5 to 10 μM leads to rapid mitochondrial membrane depolarization in pancreatic cancer cells [24] and other cell types [27]. Separate studies indicate that rottlerin also induces autophagy and activates the UPR in other cancer cell types [26, 36].

### Rottlerin reduces cellular metabolic activity in a dose-dependent manner in PaSC

**Fig 1:**
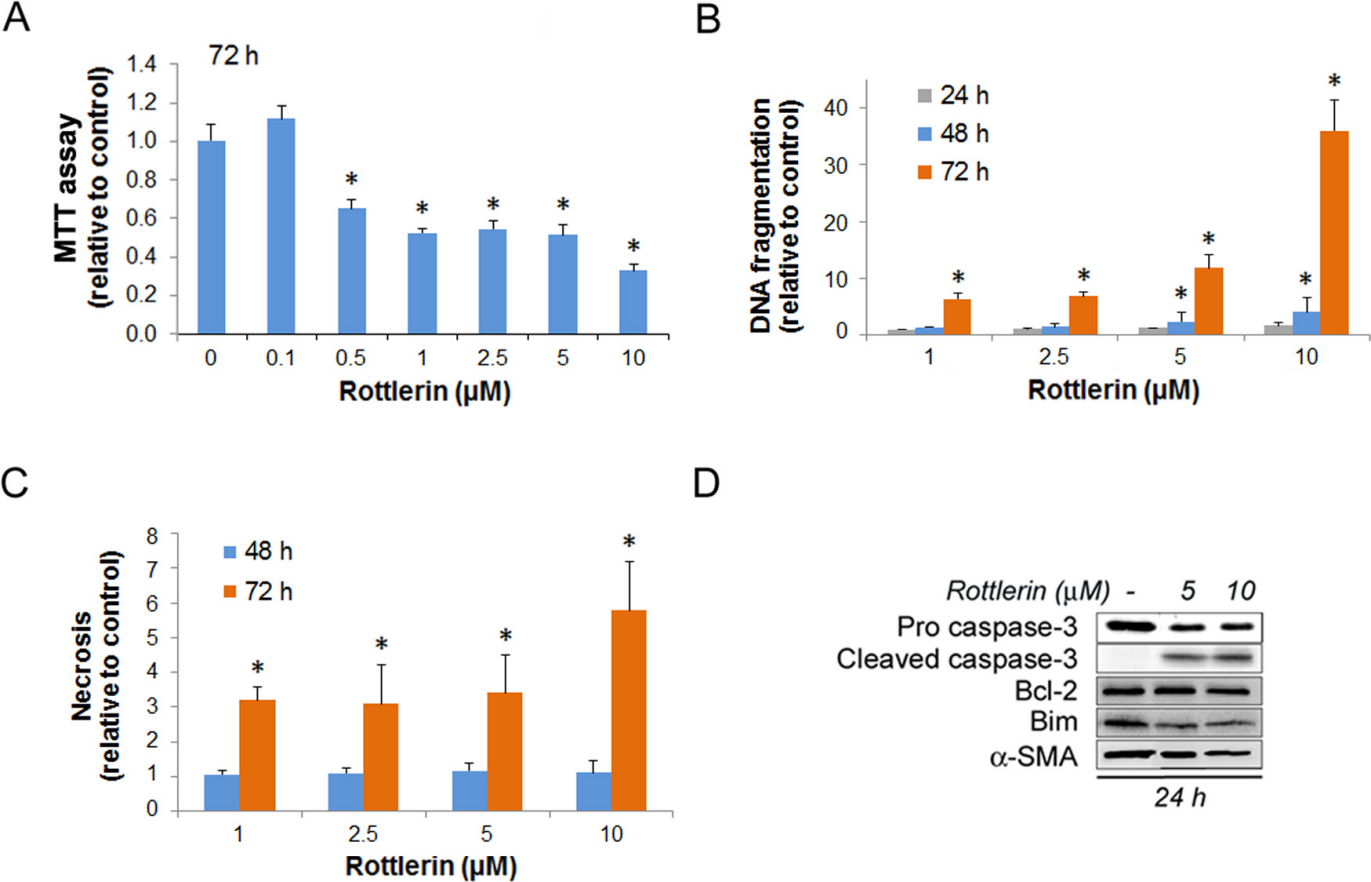
Rottlerin affects cellular metabolic state and induces apoptotic cell death. Mouse PaSC were plated in 10% FBS medium and treated with DMSO as control or rottlerin at different concentrations for up to 72 h. (A) Levels of cellular metabolic activity measured by MTT assay in mPaSC treated with rottlerin for 72 h (control O.D values were set as 1). Graph represents the mean ±SEM; n = 3. (B) Apoptosis measured by internucleosomal DNA fragmentation in cell lysates and expressed relative to controls. Graph shows mean ± SEM; n = 4. (C) Secondary necrosis measured as internucleosomal DNA fragmentation in the conditioned media and expressed relative to controls. Graph shows mean ± SEM for 2 independent experiments. Asterisks in graphs indicate statistical significance (t-test, * p<0.05 as compared to control). (D) mPaSC were treated with 5 or 10 μM rottlerin for 24 hours. Cellular levels of the apoptotic regulators caspase-3 (pro-form and active cleaved-form), Bcl2, and Bim were measured by Western blotting. α-SMA was used as a marker of PaSC activation and as loading control; immunoblot is representative of two independent experiments.

In initial studies, we determined optimal experimental conditions for induction of metabolic stress without causing premature cell death in PaSC. Primary mPaSC cultured in 10% FBS containing media were treated for 72 h with different concentrations of rottlerin, and then cellular metabolic activity was determined by MTT assay. As illustrated in Fig 1A, 0.1 μM rottlerin had little effect, but 72 h treatments with 0.5, 1, 2.5, 5 or 10 μM rottlerin reduced metabolic activity by 35, 48, 46, 49 and 64%, respectively. The potential for rottlerin to induce apoptosis were next examined at different time points.

Apoptosis as determined by internucleosomal DNA fragmentation assay was evident in PaSC treated with 1 or 2.5 μM rottlerin only after 72 h treatment (late apoptosis; Fig 1B). Compared to controls, 1 μM rottlerin increased apoptosis by 1.0-fold at 24 h and 1.4-fold at 48 h; and 2.5 μM rottlerin increased apoptosis by 1.1-fold at 24 h and 1.5-fold at 48 h, but these increases did not reach statistical significance. In contrast, rottlerin at 5 or 10 μM induced significant apoptosis within 48 h, with increases of 2.4-fold and 4.1-fold, respectively (early apoptosis). At the 72 h time point, we observed apoptosis at all 4 concentrations tested (1–10 μM). At 72 h, rottlerin also induced some late necrosis as determined by the presence of internucleosomal DNA fragments in the conditioned media (Fig 1C). However, rottlerin treatments did not induce necrosis at earlier time points, as determined by the presence of DNA fragmentation in conditioned media (Fig 1C) and by the release of lactate dehydrogenase (LDH) (not shown).

Since Bcl2 and the proapoptotic BH3-only protein Bim regulate rottlerin-induced apoptosis in other cell types [24], we measured protein expression levels of these proteins in rottlerin-treated cells. At 5 or 10 μM rottlerin, concentrations that induced early apoptosis, we observed increased levels of cleaved caspase-3 but only minor changes in Bcl2 or Bim protein levels (Fig 1D). As expected, we did not observe cleaved caspase-3 or changes in Bcl2 or Bim in cells treated with 1 or 2.5 uM rottlerin for 24 or 48 h (not shown). Thus, changes in expression levels of these anti- and proapoptotic proteins cannot account for the rottlerin-induced apoptosis obtained in these experiments. Based on the data illustrated in Fig 1, for subsequent studies using primary mPaSC we chose mainly rottlerin concentrations (1–2.5 μM) that do not induce significant cell death within 48 h. Immortalized mouse PaSC (imPaSC) are slightly more resistant to metabolic disturbances (data not shown). Consequently, we used rottlerin at 1–5 μM concentrations for experiments involving imPaSC.

### Rottlerin induces mitochondria dysfunction and reduces cellular ATP levels

**Fig 2:**
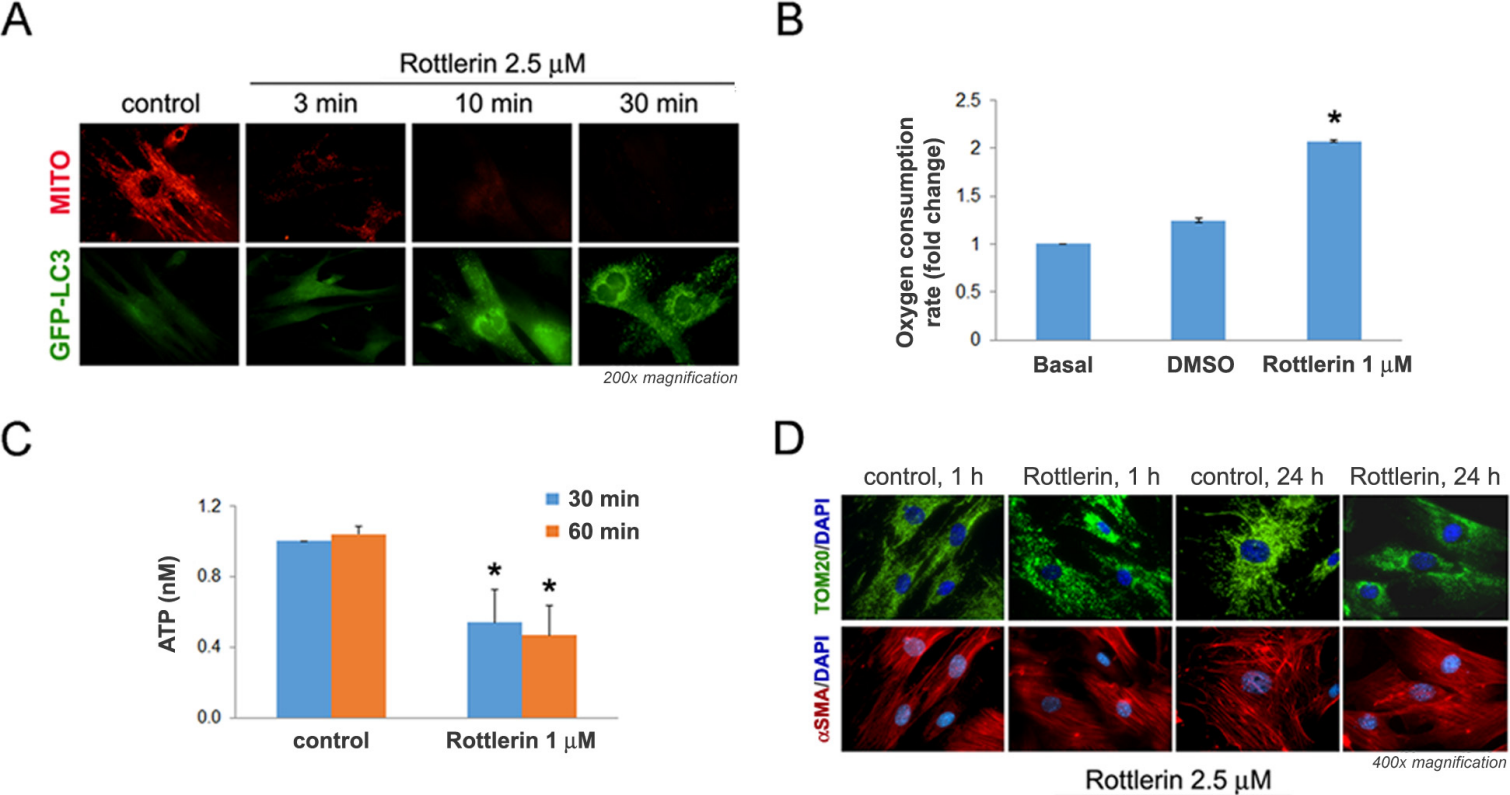
Rottlerin induces rapid mitochondrial dysfunction in mPaSC that precedes LC3 puncta formation. (A) mPaSC isolated from GFP-LC3 transgenic mice were labeled with 25 nM MitoTracker (MITO; red fluorescence) for 10 min and then treated without (control) or with 2.5 μM rottlerin for the indicated times. MITO staining (red) and GFP-LC3 puncta (green fluorescence) formation in live cells were visualized using a fluorescence microscope. (B) Rate of cellular oxygen consumption measured in live imPaSC under basal conditions and after sequential additions of DMSO and 1 μM rottlerin. Data is expressed as fold change relative to basal. Graph shows mean ± SEM; 3 independent experiments; * p<0.05 as compared to basal (t-test). (C) mPaSC were treated with 1 μM rottlerin for 30 or 60 minutes and total cellular ATP levels were measured in cell lysates by luminescence assay. Graph shows mean ± SEM for 3 independent experiments; * p<0.05 as compared to control (t-test). (D) mPaSC treated with 2.5 μM rottlerin for 1 or 24 hours were double immunostained for the mitochondrial marker Tom20 (green staining) and the stellate cell marker α-SMA (red staining); nuclei were counter-stained with DAPI (blue staining). Images were visualized under fluorescence microscope.

Rottlerin was initially identified as a protein kinase inhibitor with some specificity for the Protein Kinase C isoform delta (PKC-δ) [37]. However, later studies convincingly demonstrated that rottlerin acts mainly as a mitochondria uncoupler with poor selectivity for PKCs [27], although the precise molecular target(s) for its mitochondrial uncoupling effects has not been unambiguously identified. To determine whether rottlerin at the selected concentrations decreases mitochondrial membrane potential, and simultaneously to assess autophagy induction, we used mPaSC isolated from wild type or GFP-LC3 transgenic mice, which express GFP-LC3 fusion protein and are widely used to monitor autophagy. GFP-LC3 mPaSC were loaded with MitoTracker (Red CMXRos, MITO) for 10 min to visualize functional mitochondria, and then live cells treated with vehicle or rottlerin were observed by fluorescence microscopy. As illustrated in Fig 2A, MITO-labeled active mitochondria and diffuse LC3 staining were visualized in all vehicle-treated cells throughout the experiment. Rottlerin at 1 μM (not shown) or 2.5 μM (Fig 2A, top panel) induced a rapid (1–3 min) dissipation of MITO fluorescence in all cells, consistent with loss of mitochondrial membrane potential, and this effect persisted for at least 30 min. Dissipation of the MITO signal preceded the formation of cytoplasmic LC3 puncta that appeared in most GFP-LC3 mPaSC as early as 5 min after rottlerin treatment (not shown), and more robustly at 30 min (Fig 2A, lower panel). In PaSC pretreated for 30 min with 2.5 μM rottlerin before MITO loading, MITO fluorescence was absent in 100% of cells. Similarly, MITO fluorescence was either absent or significantly reduced in 70% of the 24-h rottlerin pretreated cells (not shown). These data show that rottlerin induces rapid, sustained loss of mitochondrial membrane potential in PaSC. Also, as expected for an uncoupler of mitochondria respiration from oxidative phosphorylation, rottlerin acutely and significantly increased the rate of oxygen consumption during real-time measurements (Fig 2B), and reduced cellular ATP levels by 50% during fixed time (30 and 60 min) incubations (Fig 2C). These data imply that activated PaSC, even when cultured under conditions of adequate nutritional support, predominantly rely on mitochondria rather than glycolysis for maintaining ATP energy levels and ongoing energy-dependent processes.

We next assessed whether rottlerin alters mitochondrial morphology and mitochondrial mass. Opposing processes of mitochondrial fusion and fission undergo dynamic regulation of the structure of the cellular mitochondrial network, termed mitochondrial dynamics [38]. Cells under normal growth conditions have elongated, interconnected mitochondria achieved by predominant mitochondrial fusion processes. Mitochondrial inhibitors or oxidative stress increase fission and/or reduce fusion over time, leading to punctate-structured mitochondria and reduced energy metabolism [39]. Here, we used immunostaining for Tom20 to determine the mitochondrial structure and dynamics in control and rottlerin-treated PaSC. Tom20 is a receptor protein of the translocase complex of the outer mitochondrial membrane (TOM complex) involved in protein import. As illustrated in Fig 2D, in control cells mitochondria (Tom20 green staining) appeared as long, interconnected tubular, and sometimes, branched structures residing throughout the cytoplasm. In contrast, treatment with 2.5 μM rottlerin produced rapid disruption of the mitochondrial network, and redistribution of Tom20 into punctate structures, i.e., events consistent with increased fission, extensive loss of mitochondrial membrane potential and depletion of cellular energy [40]. Moreover, after 24 h rottlerin treatment Tom20 staining was significantly reduced and confined to the perinuclear area in most cells (Fig 2D), suggesting a decrease in the mitochondrial mass.

Taken together, data in Fig 2 indicate that rottlerin induces mitochondria dysfunction and compromises cellular bioenergetics in cultured PaSC, as previously reported in other cell types.

### Rottlerin promotes autophagy dysregulation in cultured PaSC

Under conditions of reduced cellular energy, autophagy promotes cell survival by providing nutrients that can be recycled for ATP generation [9]. In addition, autophagy can protect from cell death by eliminating damaged mitochondria and ER components containing toxic misfolded proteins [10]. Previous reports indicated that rottlerin alters autophagy but the precise effects on autophagic flux and regulatory proteins vary depending on the cell type [41].

In this study, rottlerin-induced loss of mitochondrial membrane potential was rapidly followed by accumulation of LC3 puncta in GFP-LC3 mPaSC, indicating that rottlerin promotes accumulation of autophagosomes in these cells (Fig 2A). Autophagosome accumulation occurs because of a rapid increase in autophagosome formation (autophagy induction), a decrease in autophagic clearance of autophagosomes by lysosomes, or both. To gain further insight into the effects of rottlerin on autophagic flux, we monitored protein levels of soluble LC3-I and the autophagic vesicle-associated form, LC3-II, as well as protein levels of sequestosome-1 (p62/SQSTM1 or p62). p62/SQSTM1 is a ubiquitin-binding, multifunctional signaling/scaffold protein that serves as link between LC3-II, autophagosomes and polyubiquitinated proteins. p62/SQSTM1-bound polyubiquitinated proteins are incorporated into autophagosomes and degraded in autolysosomes. Since p62 gets degraded in the process, changes in steady state levels of p62 over time have been used as an index of autophagic degradation. [42, 43].

**Fig 3:**
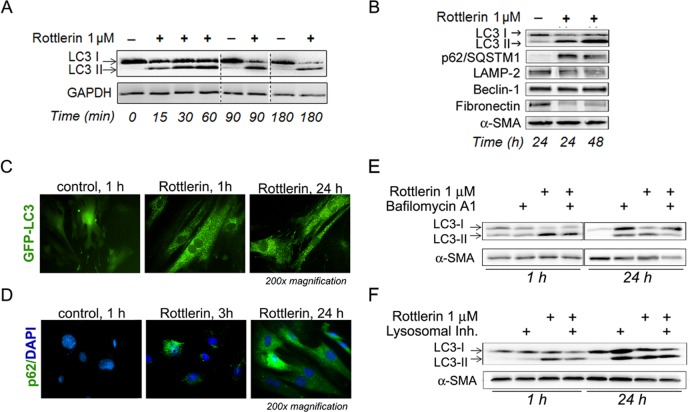
Rottlerin induces autophagy dysregulation in PaSC. (A and B) Cells were treated with vehicle (-) or rottlerin for up to 48 h. Cellular protein levels of LC3-I and its LC3-phosphatidylethanolamine conjugate (LC3-II) were analyzed by Western blotting after short (A) and long-term treatment (B). Immunoblot in panel B also shows protein levels of the autophagy regulators p62/SQSTM1, LAMP-2, and Beclin-1. Fibronectin and α-SMA levels are shown as representative markers of stellate cell activation and loading controls. (C) Live imaging of GFP-LC3 mPaSC treated with vehicle or 2.5 μM rottlerin for up to 24 h by fluorescence microscopy. Representative pictures show diffuse cytoplasmic distribution of GFP-LC3 in control cells and GFP-LC3 puncta formation upon treatment with rottlerin. (D) mPaSC were immunostained for p62/SQSTM1 (green staining) and DAPI was used for visualization of nuclei. Pictures show progressive formation of p62 aggregates in mPaSC upon rottlerin treatment. (E and F) Blockade of autophagy by lysosomal inhibitors does not affect LC3 lipidation in mPaSC. Cells were pre-incubated for 30 min with bafilomycin A1 (E) or a lysosomal inhibitor cocktail (bafilomycin A1 + pepstatin A + E-64; panel F), and then incubated for 1 or 24 h with 1 μM rottlerin. Protein levels of LC3 and α-SMA were measured by Western blotting as autophagy marker and loading controls.

Wild-type mPaSC were treated for up to 48 h with non-apoptotic rottlerin concentrations (1–2.5 μM). Western blot analysis showed marked increases in LC3-II levels and concomitant reduction in LC3-I as early as 15 min after rottlerin treatment, and this effect persisted for at least 48 h (Fig 3A and 3B). These data suggest conversion of LC3-I into LC3-II in rottlerin-treated PaSC and increased autophagic flux. Moreover, we found marked LC3 puncta formation in 90% of GFP-LC3 mPaSC after 1 h and persisting until at least 24 h post rottlerin treatment (Fig 3C). Of note, GFP-LC3 cells did not exhibit apoptotic nuclear morphologic changes during the 24h-rottlerin treatment period (not shown). Different from a previous report in cancer stem cells [44], rottlerin treatments did not alter protein levels of the autophagy regulator, beclin-1. We also found slightly decreased levels of the lysosomal membrane protein LAMP-2 (Fig 3B).

Our data showed that rottlerin markedly increased protein levels of p62/SQSTM1 (Fig 3B). Immunofluorescence analysis for p62/SQSTM1 showed progressive development of p62/SQSTM1 aggregates in rottlerin-treated cells, with 5%, 18% and 90% of cells displaying p62 puncta at 1 h, 3 h and 24 h treatment, respectively (Fig 3D). These data suggest that autophagosome degradation may be impaired in these cells, thereby accounting at least in part for the accumulation of p62/SQSTM1. However, we found that after 6 h treatment rottlerin also increased p62/SQSTM1 mRNA levels (see below). Thus, the sum total of the rottlerin-induced p62/SQSTM1 accumulation may reflect contributions of both a reduction in autophagic degradation as well as an increase in p62 mRNA expression.

To further assess rottlerin effects on autophagic flux, mPaSC were treated with lysosomal inhibitors to impair lysosomal function and block the degradation of autophagosome cargo (Fig 3E). mPaSC were pretreated with bafilomycin A1, a lysosomal vacuolar H+-ATPase (proton pump) inhibitor, to interrupt autophagosome-lysosome fusion, then with vehicle or rottlerin. Western blots of LC3-II in these cell lysates showed that there was no enhancement of rottlerin-induced increases by bafilomycin A1 pretreatment (Fig 3E), suggesting that rottlerin alone is capable of interrupting autophagic flux. In case there was residual lysosomal function when bafilomycin A1 alone was used as an inhibitor, cells were also pretreated with a cocktail of lysosomal inhibitors including bafilomycin A1, E-64d (a cysteine protease inhibitor) and pepstatin A (an aspartic protease inhibitor) before rottlerin treatment. In this case again, lysosomal inhibitor pretreatment did not enhance the levels of rottlerin-induced LC3-II observed above that obtained from rottlerin treatment (Fig 3F). Taken together, these data support the concept that rottlerin, at the low concentrations tested, induces a rapid increase in autophagic activity in PaSC (indicated by rapid conversion of LC3-I into LC3-II) that is accompanied by a blockade in the autophagic flux. The inability of rottlerin-treated cells to complete autophagy can be due to the depletion in cellular ATP, required for autophagy completion, or a direct effect of rottlerin on lysosomal function. Since functional autophagy is critical for stellate cell survival, rottlerin-induced inhibition of autophagy further compromises cellular energy status and substrate availability for anabolic processes.

### Rottlerin inhibits mTORC1 signaling in PaSC by AMPK-independent mechanisms

AMP-activated protein kinase (AMPK) is recognized as a critical regulator of cellular energy homeostasis [22]. Activation of AMPK by low cellular energy charge (ATP/AMP ratios) inhibits anabolic processes such as protein and fatty acid synthesis, activates autophagy and can modulate apoptosis signals in energetically compromised cells. AMPK actions are mediated at least in part by inhibition of mTORC1, and previous reports showed that rottlerin and other mitochondrial inhibitors such as metformin exert their effects via AMPK- and mTORC1-dependent mechanisms [28, 45, 46].

**Fig 4:**
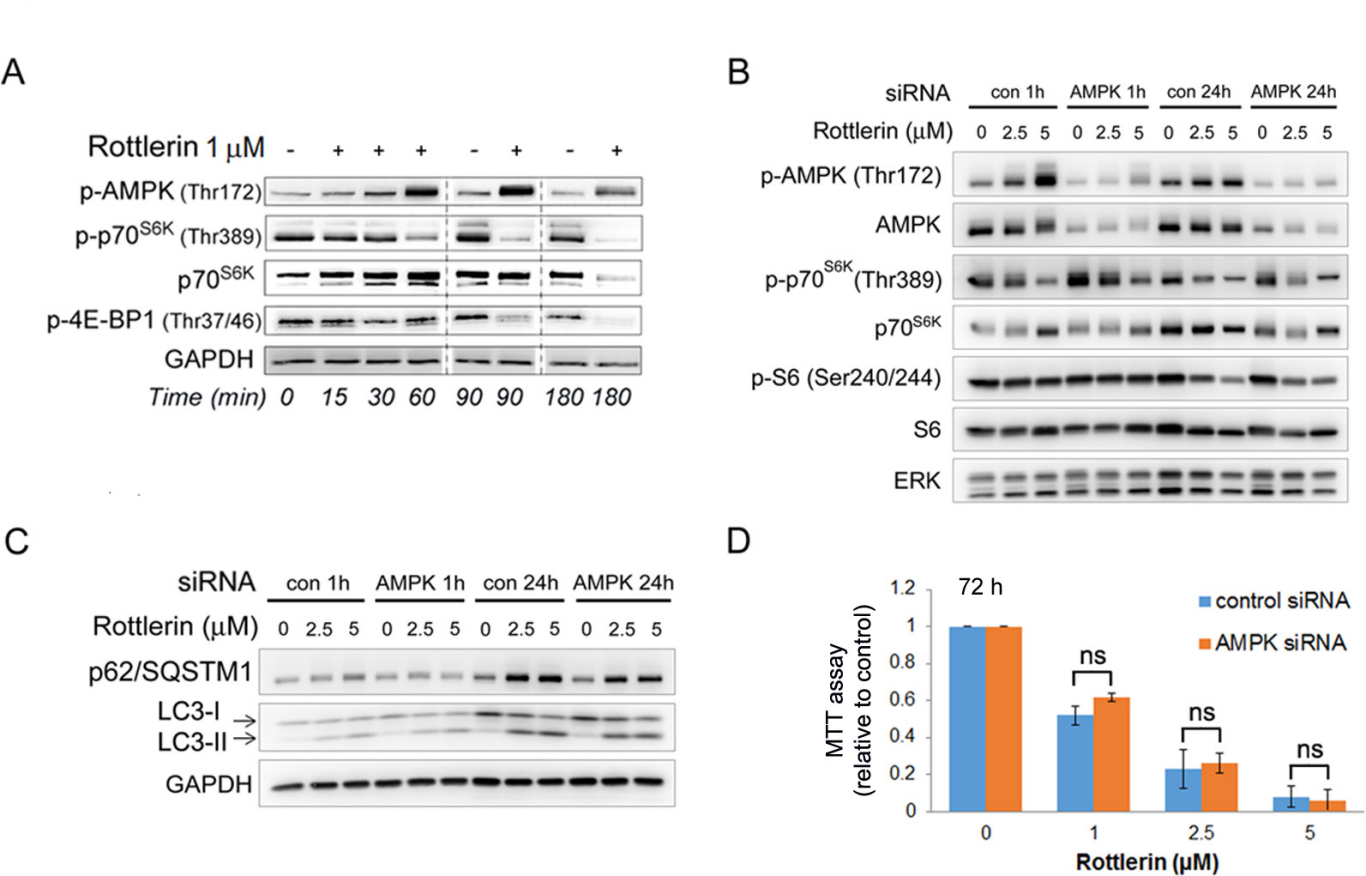
Rottlerin inhibits mTOR activation and autophagy in PaSC by AMPK-independent mechanisms. (A) Primary mouse PaSC were treated with 1 μM rottlerin and Western blotting analysis was used to determine the activation state of AMPK and the mTORC1 targets p70 S6K and 4E-BP1. (B and C) Immortalized mouse PaSC were transfected with non-targeting (“con”) or AMPKα1/2 siRNA (“AMPK”) and then treated with rottlerin at the indicated concentrations for 1 h or 24 h. (B) Levels of total and phosphorylated AMPK, p70 S6K, S6 and ERK (loading control) were measured by Western blotting. (C) Levels of the autophagic markers LC3, p62, and GAPDH (loading control) were measured by Western blotting. (D) Cellular metabolic state of mock-transfected or AMPKα1/2 siRNA transfected imPaSC treated with vehicle or rottlerin for 72 hours was assessed by MTT assay. Graph shows O.D. values relative to control cells (mean ±SEM). As indicated in the graph, the reduction in cellular metabolic state induced by rottlerin was comparable in mock- and siRNA transfected cells. Data is representative of 3 independent experiments; ns = no statistical significant differences between control and AMPK siRNA transfected cells (t-test).

To determine the participation of AMPK/mTORC1 signaling in rottlerin effects, primary or immortalized mPaSC cultured in 10% FBS containing media (17 mM glucose) were treated with rottlerin for up to 48 h. As illustrated in the immunoblots in Fig 4A and 4B, rottlerin induced a rapid and potent activation of AMPK, as shown by a significant increase in phosphorylation of AMPK α subunit at Thr172, and this effect persisted for 48 h (not shown). Moreover, rottlerin dose-dependently inhibited mTOR signaling as indicated by reduced phosphorylation of the mTORC1 substrates p70 S6 kinase at Thr389, S6 at Ser240/244, and 4E-BP1 at Thr37/46 (Fig 4A and 4B). Therefore, inhibition of mTORC1 substrates is linked to reduced cell growth and proliferation, and inhibition in protein translation.

As mentioned above, several reports showed that rottlerin exerts its effect through AMPK-dependent mechanism. To further interrogate the role of AMPK in rottlerin-induced mTOR signaling inhibition, we knocked down expression of both α1 and α2 AMPK catalytic subunits in imPaSC by transfection with specific siRNA. As shown in Fig 4B, control siRNA treated imPaSC exhibited dramatic AMPK activation in response to rottlerin, as judged by AMPK Thr172 phosphorylation, as well as potent inhibition of mTORC1, as determined by decreases in the phosphorylation state of the mTORC1 substrates p70S6K at Thr389 and S6 at Ser240/244. However, whereas siRNA mediated knockdown of AMPK dramatically attenuated the appearance of Thr172-phosphorylated (active AMPK), this was not accompanied by any reversal of the rottlerin-induced mTORC1 inhibition (Fig 4B). Moreover, siRNA treatment did not prevent rottlerin-induced accumulation of the autophagy markers p62 and LC3 (Fig 4C) or the decrease in cellular metabolic activity, as determined by MTT assay (Fig 4D). In conclusion, our data indicates that, at the doses and under the conditions tested, rottlerin inhibition of mTOR signaling and autophagy are predominantly mediated by AMPK-independent mechanisms.

### Endoplasmic reticulum stress and transcription factor CHOP modulate cell fate in rottlerin-treated PaSC

Metabolic stress or mitochondrial dysfunction is linked in many cells to activation of homeostasis pathways including autophagy and ER stress responses. The best characterized ER stress response is the Unfolded Protein Response (UPR), a complex signaling system that is activated by the presence of unfolded proteins within the ER lumen. The UPR governs ER function as well as prosurvival or proapoptotic cell fate decisions upon diverse cellular stresses. During recent years, experimental evidence has identified intense crosstalk among the UPR, autophagy and mitochondria [47]. Autophagy can get activated by reductions in cellular energy state and subsequent inhibition of mTOR signaling as well as by UPR activation [48]. Moreover, evidence supports an interaction between the UPR and mTOR that modulates autophagy activity and apoptosis, although the mechanisms underlying this interaction are not well defined yet [49].

**Fig 5:**
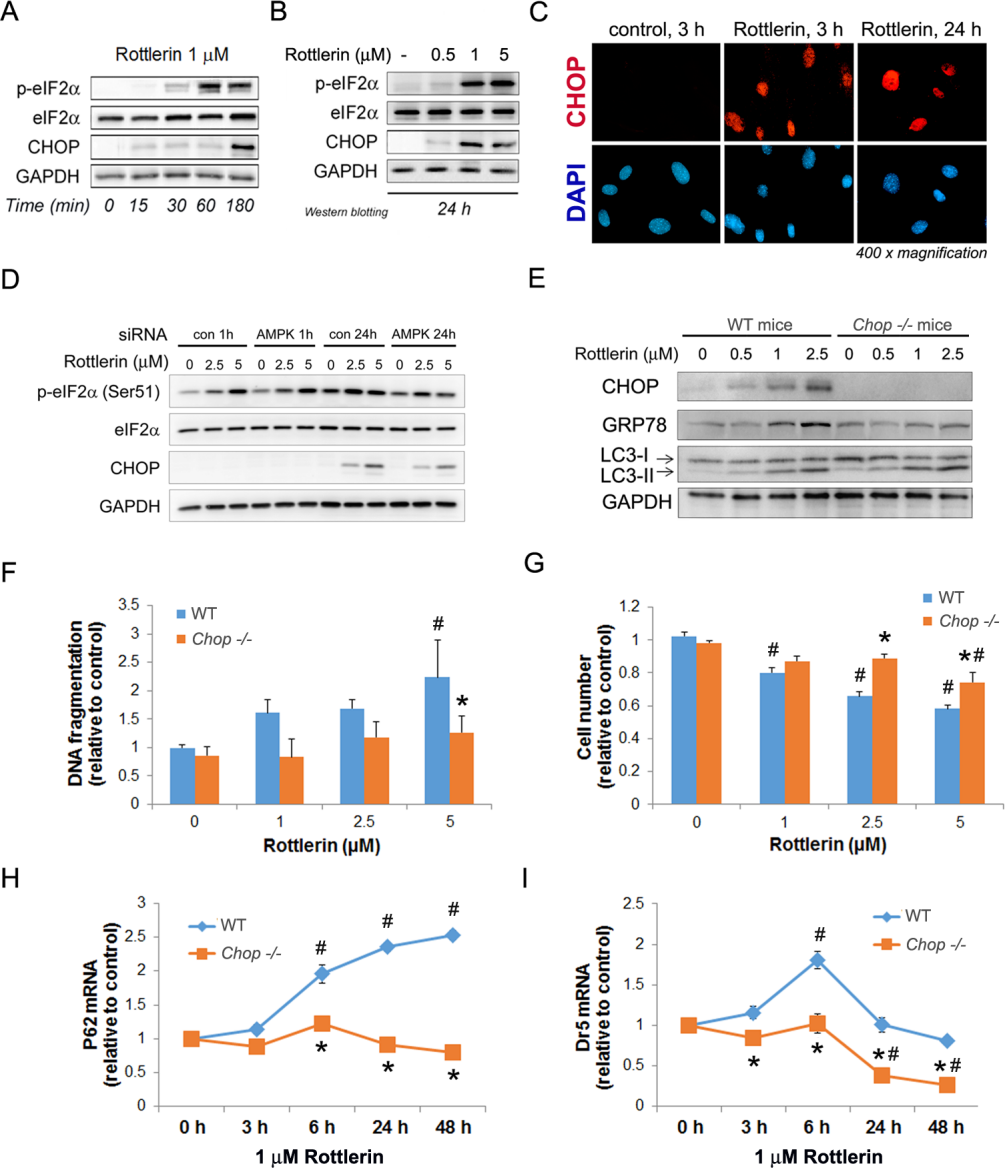
CHOP regulates cell death in rottlerin-treated PaSC. (A and B) mPaSC were treated with different concentrations of rottlerin at the indicated times. Activation of the PERK/eIF2α branch of the UPR measured by the protein levels of total and phosphorylated eIF2α and the proapoptotic transcription factor CHOP. GAPDH expression was analyzed as a loading control. As shown, CHOP upregulation could be detected as early as 15 min in cells treated with 1 μM rottlerin (A) and this effect was sustained for at least 24 h (B). (C) Immunofluorescence for CHOP (red staining) in nuclei (blue DAPI staining) of mPaSC treated with 1μM rottlerin for 3 h and 24 h. (D) AMPKα1/2 were silenced by siRNA in imPaSC and then cells treated with rottlerin at indicated concentrations for 1 h or 24 h. p-eIF2α, total eIF2α, CHOP and GAPDH (loading control) were measured by Western blotting. (E) mPaSC isolated from wild type (WT) or *Chop -/-* mice were treated with rottlerin for 24 h. Immunoblots show protein levels of CHOP, GRP78, LC3 and GAPDH (loading control). (F and G) Cell death was assessed in WT or *Chop -/-* mPaSC treated with rottlerin for 48 h. Apoptosis was determined by DNA fragmentation ELISA (panel F) and cell number (panel G). Data in graphs is presented as mean ±SEM, n = 3; * p<0.05 as compared to WT; # p<0.05 as compared to control at 0 μM rottlerin (two way ANOVA followed by post-hoc Tukey tests). (H and I) p62 (panel H) and death receptor 5 (Dr5; panel I) mRNA levels were determined by qPCR in rottlerin-treated WT and *Chop -/-* mPaSC. As indicated, rottlerin-induced upregulation of p62 and Dr5 were blunted in *Chop -/-* cells. Data in graphs is presented as mean ±SEM, n = 3–4; * p<0.05 as compared to WT; # p<0.05 as compared to time 0 (two way ANOVA followed by post-hoc Tukey tests).

Our studies indicate that rottlerin persistently activates signaling downstream of the UPR sensor PERK in mPaSC. In particular, rottlerin treatment induced robust phosphorylation of eukaryotic translation initiation factor 2 alpha (eIF2α) at Ser 51, an effect linked to general blockade of ER protein translation (Fig 5A and 5B). Moreover, rottlerin, at concentrations as low as 0.5 μM and more robustly at 1 and 5 μM, upregulated CHOP, a transcription factor that modulates ER protein translation in conditions of ER stress, and promotes ER-dependent apoptosis (Fig 5A and 5B). In rottlerin-treated PaSC, CHOP could be visualized by immunofluorescence in the nuclei of approximately 15% of cells at 1 h (not shown), 35% of cells at 3 h, and 95% of cells at 24 h treatment (Fig 5C). As we observed for mTOR signaling, AMPK knockdown did not prevent rottlerin effects on eIF2α phosphorylation or CHOP expression (Fig 5D), suggesting that AMPK does not regulate ER stress signaling in our experimental conditions.

Interestingly, compared to wild type, mPaSC isolated from CHOP deficient mice (*Chop -/-*) displayed comparable levels of LC3-II after rottlerin treatment, but reduced ER stress, as indicated by the ER stress marker GRP78 (Fig 5E). Moreover, genetic CHOP deletion significantly reduced apoptosis and increased cell survival in response to high concentrations of rottlerin (Fig 5F and 5G), supporting a key role for the UPR and CHOP in regulating cell fate decisions under metabolic stress. The mechanisms underlying CHOP proapoptotic effects are not completely understood. Proposed mechanisms include ATP depletion by promoting ATP-dependent ER protein translation in conditions of ER stress [20], dysregulation of autophagic regulators including p62/SQSTM1 [19, 41], and upregulation of cell death receptor 5 (DR5) [18, 50]. In our study, we found that genetic deletion of CHOP prevented rottlerin-induced upregulation of p62/SQSTM1 and Dr5 mRNA (Fig 5H and 5I), supporting a role for these proteins in CHOP regulation of apoptosis responses in PaSC.

### Metabolically stressed PaSC differentially regulate gene expression

**Fig 6:**
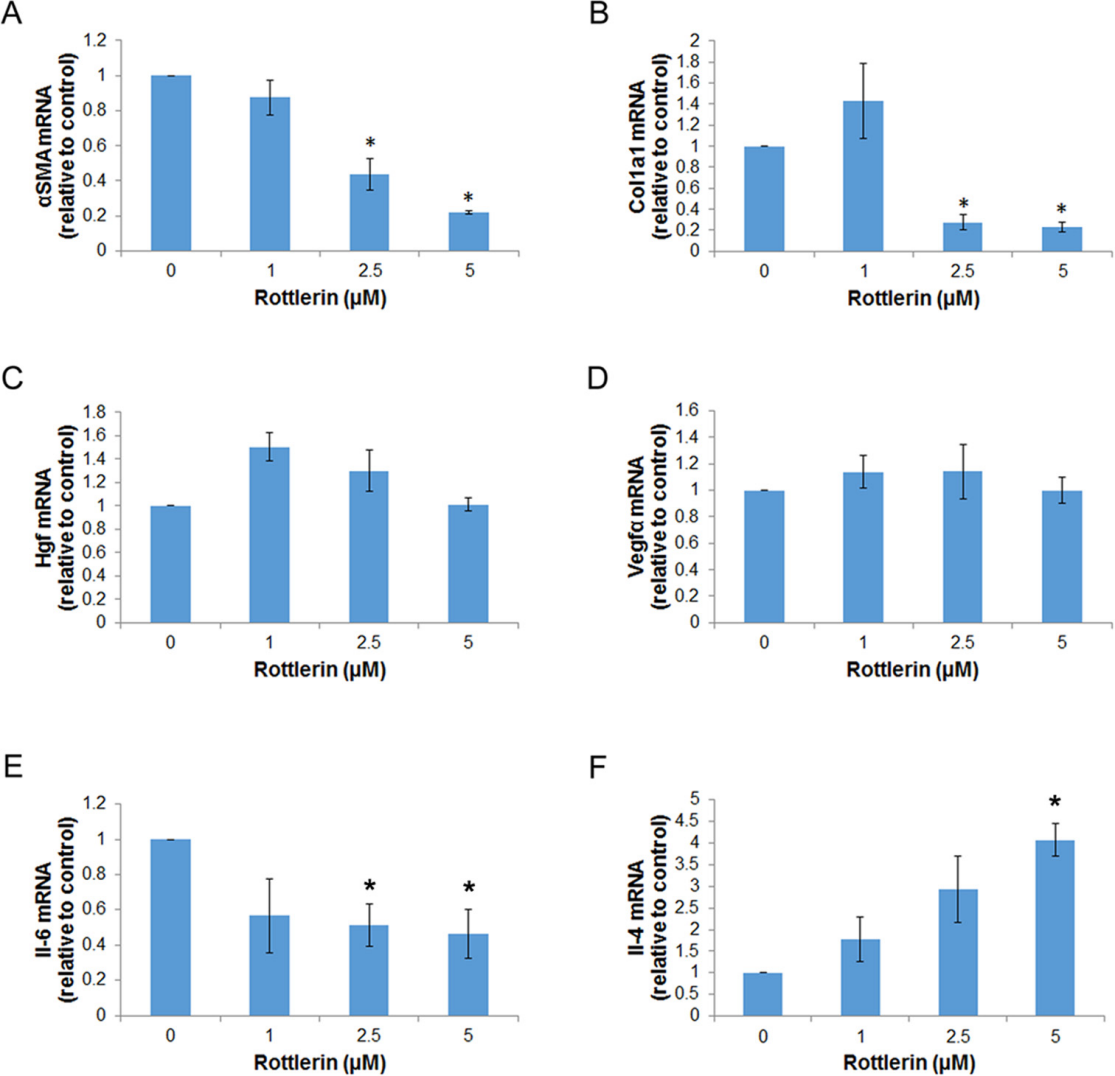
Metabolically stressed PaSC exhibit differential expression of profibrotic and inflammatory markers. imPaSC were treated with rottlerin at different concentrations for 24 h. mRNA expression of (A) *αSMA*, (B) *Col1a1*, (C) *Hgf*, (D) *Vegfα*, (E) *Il-6* and (F) *Il-4* was determined by qPCR. Data in graph is mean ±SEM from three independent experiments; * p<0.05 as compared to WT (t-test).

Activated PaSC participate in the remodeling of surrounding extracellular matrix (ECM) through deposition of collagens and other ECM components, and also exert paracrine effects on neighboring cells by the release of mitogenic factors and inflammatory cytokines [4]. To determine further the phenotype of metabolically stressed PaSC, we assessed by qPCR the expression of key factors produced by these cells. For these studies, we treated immortalized or primary mPaSC with 1, 2.5 or 5 μM rottlerin for 24 h, a time point when cells did not exhibit loss in cell viability or apoptosis (Fig 1), but did show marked inhibition of mTOR (Fig 4B) and activation of PERK/CHOP signaling (Fig 5B and 5D). Fig 6 illustrates the data obtained in imPaSC; similar results were obtained in primary mPaSC (not shown).

Our data indicate that rottlerin reduces fibrogenic potential in PaSC as indicated by decreased cellular expression of the stellate cell marker α-SMA (Fig 6A), and reduced production of the extracellular matrix (ECM) proteins collagen type I, alpha 1 (Fig 6B), the main collagen in fibrotic tissues, and fibronectin (Fig 3B). Interestingly, as shown in Fig 6C and 6D rottlerin treatment even at 5 μM concentration did not affect expression levels of the growth factors hepatocyte growth factor (HGF) and vascular endothelial growth factor alpha (VEGFα). Both HGF and VEGFα have angiogenic properties in pancreatic cancer and promote tumor growth and invasion [51, 52]. Studies in cardiomyoblasts showed that VEGFα expression was unresponsive to metabolic stress inhibition due to enhanced mRNA stability in conditions of stress [53]. We have not assessed whether a similar mechanism is responsible for preservation of HGF or VEGFα mRNA expression in rottlerin-treated PaSC, but a sustained production of these growth factors by PaSC may support angiogenesis in the tumor microenvironment even in conditions of hypoxia and metabolic stress. We can speculate that in conditions of hypovascularization, metabolically stressed PaSC would decrease the production of ECM proteins but they would retain their capacity to support angiogenic processes that alleviate hypoxia and poor substrate availability and fuel tumor progression.

Cytokines are important intercellular mediators and autocrine factors within the inflammatory microenvironment due to their ability to support biological responses in their target cells. Accordingly, we also measured expression levels of IL-6, IL-4 and IL-13 generated by PaSC. IL-6 signaling has been implicated in various cancers, as the IL-6 receptor complex is linked to JAK activity targeting STAT3. IL-4 has roles in immune cell modulation. Different from signaling by the IL-6 receptor, that mediated by the IL-4 receptor is mainly propagated via STAT6. As determined by qPCR, IL-13 mRNA levels were very low in both control and rottlerin treated cells (not shown). By contrast, IL-6 expression was robust in control cells, although it decreased by 2-fold in rottlerin-treated cells (Fig 6E). Surprisingly, we found and show here for the first time that expression levels of IL-4 increased by rottlerin treatment in a dose dependent manner (Fig 6F). These data indicate that, compared with IL-6 production, IL-4 is more readily expressed under conditions of low cellular energy. This implies in general that cytokine production is differentially regulated in PaSC under conditions of low cellular energy and ER stress, with some cytokines including IL-4 being preferentially up-regulated. This data may have further significance, since IL-4 has been implicated in immunoregulation of pancreatic fibrosis via PaSC [54]. In particular, a recent study implicated PaSC as the source of IL-4 as an essential factor inducing alternatively activated macrophages [54].

## Discussion

Activated pancreatic stellate cells are established as the central cell type responsible for generating stroma, but the effects of metabolic perturbation that may lead to differential pro- or anti-tumorigenic effects on these cells remain largely unexplored. To selectively impair mitochondrial function and assess its consequences, we selected rottlerin, a natural product with a long history of use in traditional medicine and with some promise as an anti-tumorigenic agent [55, 56].

To selectively observe changes, rottlerin doses were titrated to levels that did not produce significant cell death, even at extended incubations of 48 h. Previous studies showed that rottlerin impairs mitochondrial function in several cell types [24, 26, 27], but this effect was not previously examined in activated PaSC. Our data document convincingly that low concentrations of rottlerin (1–2.5 μM) abolished mitochondrial membrane potential and led to dramatic restructuring of mitochondria from a widespread, interlocked network, first to dispersed puncta and then to a disorganized, diminished mass localized to the perinuclear region. These data confirmed mitochondrial alterations as an early event in response to rottlerin treatment in PaSC.

We then examined the loss of cellular energy in PaSC and its effects on major regulatory signaling pathways. ATP was depleted rapidly and AMPK was activated as shown in other studies with rottlerin in other cell types [28, 29]. In rottlerin-treated PaSC, mTOR activity was also diminished, and autophagy appeared to be stimulated at the level of regulatory control by mTOR, but also blocked at the level of autolysosome resolution. This autophagic blockade and the mTOR inactivation were not removed by siRNA-mediated knockdown of both (α and β) isoforms of AMPK, suggesting that AMPK-independent pathways contribute to the overall inhibition of mTOR as well as autophagy induction and downstream impairment. Consistent with these observations, Kalender et al [57] reported that in mouse embryonic fibroblasts metformin, an anti-diabetic drug that decreases mitochondrial oxidative phosphorylation, inhibits mTORC1 via Rag GTPases rather than via AMPK activation, and similar AMPK-independent effects were reported for low concentrations of metformin in pancreatic cancer cells [46].

mTORC1, by integrating nutrient and hypoxia signaling is an important repressor of autophagy and a key regulator of protein synthesis and growth. Emerging evidence also supports a critical role for mTORC1 and autophagy in fibroblast reprogramming and plasticity [11], an effect modulated by accumulation of p62. Our data shows that rottlerin-induced mitochondrial impairment led to significant increase in p62 protein levels and formation of p62 protein aggregates, effects linked to diminished turnover as well as an increase in steady state p62 mRNA levels. We have not assessed whether direct rottlerin effects on mTORC1 and/or p62 modulate metabolic reprograming in PaSC, but our data associate inactivation of mTORC1 and p62 accumulation with reduced fibrogenic potential and differential transcriptional regulation of inflammatory cytokines, results that have important implications due to the pleiotropic effects of PaSC in the tumor stroma. We found that rottlerin decreased expression of IL6, a potent protumorigenic cytokine, but increased expression of IL4, an important immune modulator of activated T cells and macrophages. Interestingly, p62 deficiency in stromal fibroblasts has been associated with overexpression of IL6 and promotion of prostate tumorigenesis [58], supporting a role for p62 in regulating inflammatory responses in the tumor stroma.

In this study, we also examined ER stress pathways to determine their involvement in the intermediate- and long-term effects of mitochondrial impairment. We found that activation of the eIF2α-CHOP pathway was dramatically upregulated by rottlerin, and cell death in response to rottlerin was diminished in *Chop -/-* PaSC. These data establish that CHOP induction is important in the cellular fate response to mitochondrial impairment in PaSC. We also found that rottlerin-induced p62 overexpression was blunted in *Chop -/-* PaSC, a result consistent with previous reports demonstrating that CHOP can directly activate transcription of p62 and other autophagic regulators [19]. Interestingly, Goodall et al [59] reported that CHOP differentially regulates the profile of cytokines produced in dendritic cells. In particular, they found that CHOP specifically upregulated IL23 expression in LPS-treated monocyte-derived dendritic cells without altering expression levels of IL8, IL1β or CCL-3. We have not assessed yet the role of CHOP in the secretory profile of rottlerin-treated PaSC, but it is tempting to speculate that PERK/CHOP signaling can modulate inflammatory responses in PaSC via p62 regulated pathways or via direct transcription of specific inflammatory cytokines.

In sum, our data indicate that metabolically stressed PaSC modulate pro-fibrogenic and proinflammatory responses in a different manner than non-stressed PaSC, and imply that such phenotypic changes affect the dynamics of pancreatic fibrosis development or tumor progression. PaSC within the microenvironment of chronic pancreatitis and pancreatic adenocarcinoma have been reported to have pro-tumorigenic effects [4]. However, more recently the pro-tumorigenic role of PaSC has been questioned and evidence presented for an anti-tumorigenic properties of this cell type [60, 61]. It therefore seems that PaSC can exhibit distinct phenotypes, perhaps in response to specific environmental changes and nutrient availability in the tumor microenvironment. For example, PaSC responses may vary regionally in the tumor depending on their proximity to well vascularized areas or hypovascularized, hypoxic areas. They can also vary temporally during tumor progression. Presumably, the profile or spectrum of cytokines secreted by the affected cells is an important and pivotal determinant of their overall pro- or anti-tumorigenic roles. Effective reprogramming to a nontumorigenic phenotype can be achieved as a therapeutic goal, only when appropriate stimuli have been identified and characterized. Further studies are needed to resolve the full range of activities of PaSC under different conditions and the potential for therapeutic intervention.
